# Hypouricemia in Behçet’s Syndrome: Prevalence and Clinical Outcomes

**DOI:** 10.3390/medicina61040739

**Published:** 2025-04-17

**Authors:** Burak Oz, Ibrahım Gunduz, Ahmet Karatas, Suleyman S. Koca

**Affiliations:** 1 Department of Rheumatology, Firat University Faculty of Medicine, 23200 Elazig, Turkey; boz@firat.edu.tr (B.O.); kocassk@yahoo.com (S.S.K.); 2 Department of Rheumatology, Selahaddin Eyyubi State Hospital, 21100 Diyarbakir, Turkey; abrahim724gunduz@hotmail.com

**Keywords:** Behçet’s syndrome, uric acid, hypouricemia, mortality

## Abstract

*Background and Objectives*: Behçet’s syndrome (BS) is a systemic inflammatory disorder characterized by recurrent oral and genital ulcers, uveitis, and vascular involvement. Serum uric acid (SUA) has been implicated in various inflammatory conditions, due to its antioxidant properties and role in oxidative stress. Abnormal SUA levels, particularly hypouricemia, may influence inflammatory processes, but their significance in BS pathophysiology remains unexplored. This study aimed to determine the prevalence of abnormal SUA levels among BS patients and investigate their associations with its clinical manifestations and laboratory parameters. *Materials and Methods*: A retrospective analysis was conducted on 436 patients with complete data who met the international criteria for Behçet’s syndrome, including 420 patients classified as hypouricemic or normouricemic, for detailed evaluation. Patients were classified as hypouricemic (<3 mg/dL), hyperuricemic (>7 mg/dL), or normouricemic (3–7 mg/dL). Data on sociodemographics, laboratory findings, and clinical characteristics were collected. Mortality and malignancy associations were analyzed using logistic regression. Inverse probability weighting (IPW) was employed to adjust for confounding factors. *Results*: Initial unadjusted analysis showed that hypouricemic BS patients had significantly lower rates of acneiform lesions (7.3% vs. 14.4%, *p* = 0.020) and vascular involvement (3.8% vs. 11.6%, *p* = 0.038) compared to normouricemic patients. However, after adjustment for confounding variables using the IPW methodology, these associations lost statistical significance (*p* = 0.592 and *p* = 0.519, respectively). Both before and after adjustment, no significant differences were observed between groups regarding major organ involvement, disease severity, or activity markers. *Conclusions*: After controlling for confounding factors, hypouricemia in BS patients did not demonstrate significant associations with specific clinical manifestations or disease outcomes. While the unadjusted data initially suggested potential relationships with acneiform lesions and vascular involvement, these associations were not supported by comprehensive statistical analysis. Further prospective studies are warranted to elucidate the complex relationship between uric acid metabolism and BS pathophysiology.

## 1. Introduction

Behçet’s syndrome (BS) is a chronic multisystemic inflammatory disease characterized by various clinical manifestations, including recurrent oral and genital ulcers and uveitis. Although the exact etiology of the disease remains unclear, it is increasingly recognized that many factors, including infections, genetic predisposition, and immune system dysfunction, contribute to its etiopathogenesis [1].

Uric acid, a byproduct of purine metabolism, plays a multifaceted and complex role within the body, exhibiting both pro-inflammatory and anti-inflammatory properties that have garnered significant research attention in recent years [2]. High serum uric acid (SUA) levels (hyperuricemia) are often linked to gout and other inflammatory conditions, while low levels (hypouricemia) can also have clinical significance. It has been shown that the inflammatory process in acute gout arthritis leads to a decrease in SUA concentrations; this indicates a complex interplay between inflammation and uric acid metabolism. The role of uric acid in inflammation is multifaceted. It has been shown that uric acid can activate the NLR family pyrin domain containing 3 (NLRP3) inflammasome, a critical component of the innate immune response, leading to the production of pro-inflammatory cytokines. This mechanism may be particularly important in BS, where systemic inflammation is a hallmark of the disease. Elevated uric acid levels have been associated with increased inflammatory markers, suggesting that uric acid might contribute to the inflammatory environment in patients with BS. Furthermore, the dysregulation of immune cells induced by uric acid has been proposed as a mechanism for inflammation associated with hyperuricemia and its complications [3,4,5,6]. A number of studies have indicated the potential for both hyperuricemia and hypouricemia to occur in patients with BS, with the possibility that these states may influence disease outcomes in divergent ways. For instance, hypouricemia has been associated with an elevated mortality rate in specific conditions, suggesting a potential adverse impact of low uric acid levels on health. In patients with BS, the presence of hypouricemia could be indicative of a more severe disease state or a distinct metabolic disorder that necessitates further investigation. This is of particular importance, given the established connection between uric acid and cardiovascular risk factors, which is a major concern in the management of BS. The interplay between uric acid levels and inflammatory processes in BS gives rise to significant questions regarding the underlying mechanisms involved. For instance, the activation of the nuclear factor kappa B (NF-κB) signaling pathway by uric acid has been associated with the promotion of inflammation, which may exacerbate the clinical manifestations of BS [7,8].

The presence of comorbidities, such as cardiovascular disease, has the potential to further complicate the relationship between uric acid and BS, given the shared inflammatory pathways inherent to both conditions. The relationship between BS and uric acid levels is intricate and merits further investigation. Although evidence suggests that uric acid may contribute to the inflammatory processes associated with BS, the clinical implications of altered uric acid levels in these patients have not yet been fully clarified [9,10].

The primary objective of this study was to ascertain the prevalence of hypouricemia and hyperuricemia among patients diagnosed with BS. Furthermore, the study sought to examine the associations between hypouricemia and a range of factors, including sociodemographic characteristics, laboratory parameters, clinical manifestations, and mortality outcomes. In order to mitigate potential confounding factors and the selection bias intrinsic to observational study designs, we employed inverse probability weighting (IPW), a robust statistical method enhancing causal inference, to strengthen the validity and reliability of our findings.

## 2. Materials and Methods

The present study has been designed in a retrospective manner and approval was obtained from the Scientific Research Ethics Committee of the university (Date-No: 01.11.2024-28570) prior to the commencement of research. The study was conducted in accordance with the ethical principles and rules of the Declaration of Helsinki (2013). Patients who made follow-up visits to our Rheumatology outpatient clinic between 2010 and 2024 were retrospectively screened through the hospital automation system and patient medical records. A total of 679 patients diagnosed with BS were initially identified. Among these patients, individuals with sufficient clinical and laboratory data, those who had not received medications known to influence SUA levels (such as diuretics, allopurinol, febuxostat, or uricosuric agents), and those who fulfilled the International Criteria for Behçet’s Disease (ITR-ICBD) [11] were included for further analysis.

SUA levels were evaluated longitudinally, utilizing sequential measurements obtained after the initial diagnosis. To ensure consistency and reliability, SUA measurements were required to have been recorded at least three times, with intervals of no less than three months between each measurement. Based on these criteria, eligible patients were classified according to their SUA status for subsequent comparative analysis.

The study population consisted of 42 patients diagnosed with hypouricemia (SUA < 3 mg/dL), 16 patients with hyperuricemia (SUA > 7 mg/dL), and 376 patients exhibiting normal SUA levels (3–7 mg/dL). Due to the insufficient number of hyperuricemic patients, this group was excluded from statistical analysis. The a priori power analysis using G*Power version 3.1.9.2 (Faul, F., Erdfelder, E., Lang, A.-G., & Buchner, A.; freely available software developed at the Universität Kiel, Kiel, Germany) indicated a minimum required sample size of 64 patients per group (α = 0.05, power = 0.80, d = 0.50). Although our hypouricemic group (n = 42) was smaller, it accurately represented the real-world prevalence observed in our cohort (n = 436). To manage this limitation and the significant group imbalance, we implemented IPW, creating a balanced pseudo-population and enhancing statistical validity. Data regarding sociodemographic, laboratory, and clinical features, malignancy history, and mortality outcomes were obtained from medical records and hospital databases.

Statistical analyses were performed using Jamovi 2024 (version 2.6). The normality of the data was assessed using the Shapiro–Wilk test and variance homogeneity was assessed with Levene’s test. Continuous variables were compared between groups using either an independent sample *t*-test or Welch’s test, based on distribution. Categorical variables were summarized as frequencies and percentages, with comparisons made via chi-square or Fisher’s exact tests, as appropriate. Factors associated with mortality were identified through univariate analyses, followed by a multivariate logistic regression model using backward stepwise (Wald) selection. Statistical significance was set at *p* < 0.05. To further address confounding and selection bias, propensity scores were calculated using logistic regression (covariates: age, gender, disease duration, CRP, ESR, acneiform lesions, and vascular involvement), and IPW analysis was conducted. Group balance post-weighting was verified using standardized mean differences (SMD < 0.15, indicating good balance). IPW-adjusted associations between SUA levels and clinical outcomes were evaluated with weighted regression models, enabling robust causal inference.

## 3. Results

A total of 436 patients diagnosed with BS who met the ITR-ICBD were included in the study. Among these, 42 patients (9.6%) were classified as hypouricemic, 16 patients (3.7%) as hyperuricemic, and 378 patients (86.7%) as normouricemic. Due to the small sample size of the hyperuricemic group, statistical analyses focused on the hypouricemic and normouricemic groups.

### 3.1. Sociodemographic Characteristics

Hypouricemia was significantly more prevalent among females (17.3%) compared to males (2.4%) (*p* < 0.001). Although the mean age of patients with hypouricemia was slightly lower than that of normouricemic patients, this difference did not reach statistical significance. The observed female predominance and tendency towards younger age in the hypouricemic group may suggest underlying hormonal factors or genetic predispositions influencing uric acid metabolism. Estrogen, known to enhance renal uric acid excretion, might partly explain the notable gender disparity observed. Notably, females constituted the vast majority (88.1%) of the hypouricemic cohort in this study (Table 1).

### 3.2. Laboratory Findings

Hypouricemic patients had significantly higher neutrophil percentages (68.2% vs. 62.9%; *p* < 0.001) and lower lymphocyte counts (1610 ± 531 vs. 2194 ± 764/μL; *p* < 0.001). This resulted in an elevated neutrophil–lymphocyte ratio (NLR) (3.54 ± 1.45 vs. 2.89 ± 1.89; *p* = 0.03), suggesting a heightened inflammatory state. Mean hemoglobin levels were significantly lower in hypouricemic patients (12.6 ± 1.81 g/dL vs. 13.8 ± 1.77 g/dL; *p* < 0.001), indicating a potential link between hypouricemia and anemia. Alanine transaminase (ALT) levels were lower in hypouricemic patients (14.0 ± 1.28 U/L vs. 19.0 ± 1.08 U/L; *p* < 0.001), possibly reflecting reduced oxidative stress and hepatocyte damage (Table 1).

### 3.3. Clinical Features

Initially, patients with hypouricemia appeared to have significantly lower incidences of acneiform lesions (7.3% vs. 14.4%; *p* = 0.020) and vascular involvement (3.8% vs. 11.6%; *p* = 0.038) compared to patients with normouricemia. However, after adjusting for confounding variables using IPW, these associations lost their statistical significance (acneiform lesions: 46.7% vs. 53.3%, *p* = 0.592; vascular involvement: 30.0% vs. 23.3%, *p* = 0.519). Consequently, the initially observed differences are likely attributable to baseline demographic and clinical confounders, rather than reflecting a direct biological effect of SUA concentrations. Furthermore, the prevalence rates of oral aphthae, genital ulcers, erythema nodosum, and gastrointestinal involvement did not differ significantly between the hypouricemic and normouricemic groups (Table 2).

### 3.4. Mortality

In the unadjusted analysis, hypouricemia was significantly more prevalent among deceased patients compared to survivors (33.3% vs. 9.5%; *p* = 0.018). However, after applying IPW to balance the baseline characteristics, mortality rates were identical between the hypouricemic and normouricemic groups (both 3.33%; *p* = 1.000). Univariate logistic regression initially indicated an association between low SUA levels and mortality (OR: 0.21; 95% CI: 0.05–0.87; *p* = 0.032); nevertheless, this relationship did not persist following IPW-adjusted multivariate analysis (OR: 1.32; 95% CI: 0.23–7.54; *p* = 0.754). Conversely, in the multivariate analysis prior to IPW adjustment, a history of malignancy (OR: 55.76; 95% CI: 6.66–466.67; *p* < 0.001), chronic kidney disease (OR: 13.57; 95% CI: 1.37–133.81; *p* = 0.026), and gastrointestinal involvement (OR: 11.29; 95% CI: 1.63–77.83; *p* = 0.014) emerged as significant independent predictors of mortality. Following IPW adjustment, advanced age (OR: 1.08; 95% CI: 1.02–1.15; *p* = 0.013), the presence of vascular involvement (OR: 4.25; 95% CI: 1.05–17.18; *p* = 0.042), and elevated C-reactive protein (CRP) levels (OR: 1.04; 95% CI: 1.01–1.08; *p* = 0.023) were confirmed as significant predictors of increased mortality risk (Table 3).

## 4. Discussion

The present study highlights the notable prevalence of hypouricemia in patients diagnosed with BS, reported at a rate of 9.6%. This figure is significantly higher than that found in the general population, thus underscoring the potential relevance of uric acid metabolism in the context of this complex inflammatory disease. This finding is consistent with prior research on rheumatological conditions, where hypouricemia has been documented at rates of approximately 10.6% in treated rheumatoid arthritis patients [12]. Such a high prevalence suggests that hypouricemia may be a common feature among various inflammatory conditions, warranting further investigation into its clinical implications.

The increased prevalence of hypouricemia in BS may be attributed to a combination of hormonal influences and genetic predispositions that affect uric acid metabolism. Specifically, estrogen has been identified as a factor that enhances the renal clearance of uric acid, potentially leading to lower SUA levels [13].

This is particularly relevant in the present study, where a substantial majority (88.1%) of hypouricemic patients were women, and the hormonal effects of estrogen, which are especially pronounced in premenopausal women, may help explain the higher rates of hypouricemia observed in this demographic within the BS cohort. Furthermore, the study emphasizes a significant association between hypouricemia and inflammatory markers, suggesting that the inflammatory processes that are characteristic of BS may also play a role in influencing uric acid metabolism [14,15].

Beyond hormonal factors, this study reveals a significant association between hypouricemia and inflammatory markers, particularly noting that hypouricemic patients exhibited higher neutrophil percentages and lower lymphocyte counts, leading to an elevated NLR, indicating a heightened inflammatory state [16,17].

This finding is consistent with previous studies that have documented increased neutrophil activity and reduced lymphocyte presence in hypouricemic patients across various conditions. The inflammatory milieu of BS may, thus, contribute to the altered uric acid metabolism observed in these patients, suggesting that hypouricemia could serve as a marker of systemic inflammation [18].

The relationship between uric acid levels and inflammatory responses is likely complex. While uric acid functions as an antioxidant at normal levels, its deficiency may increase oxidative stress and exacerbate inflammation, potentially worsening the clinical manifestations of BS [12].

Additionally, this study reports that hypouricemic patients exhibited lower hemoglobin levels, indicating a potential relationship between hypouricemia and anemia. Previous research has described a U-shaped relationship between SUA levels and anemia, where both low and high levels are associated with hematological abnormalities [13,18].

This relationship may be mediated by oxidative stress and nutritional deficiencies, which tend to be more pronounced in individuals with lower SUA levels. This necessitates further investigation into the impact of uric acid on erythropoiesis and overall hematological health in the context of BS [19].

The present study initially found that certain clinical features associated with BS, including acneiform lesions and vascular involvement, appeared to occur at significantly lower rates in patients with hypouricemia compared to those with normouricemia (acneiform lesions: 7.3% vs. 14.4%, *p* = 0.020; vascular involvement: 3.8% vs. 11.6%, *p* = 0.038). This observation initially suggested the hypothesis that lower uric acid levels might exert a protective effect against specific inflammatory manifestations of the disease. Previous findings in the literature have indicated that uric acid can function as a pro-inflammatory stimulus and activate immune responses that contribute to vascular damage and cutaneous lesions [20]. However, after rigorous adjustment for confounding variables using the IPW methodology, these associations lost their statistical significance (acneiform lesions: 46.7% vs. 53.3%, *p* = 0.592; vascular involvement: 30.0% vs. 23.3%, *p* = 0.519). This indicates that the initially observed differences were likely attributable to baseline demographic and clinical confounders, rather than reflecting a direct biological relationship between SUA levels and these clinical manifestations. Therefore, while the unadjusted data appeared to suggest potential protective effects, our comprehensive statistical analysis does not support this conclusion, emphasizing the importance of controlling for confounding variables when evaluating clinical associations in complex inflammatory disorders such as BS.

However, this protective effect does not appear to extend to all clinical features, as the rates of oral aphthae, genital ulcers, and gastrointestinal involvement were similar between hypouricemic and normouricemic groups, indicating that the relationship between uric acid levels and the specific clinical manifestations of BS is not uniform [21].

The relationship between hypouricemia and mortality in BS patients is of particular interest. While a higher proportion of deceased patients exhibited hypouricemia compared to survivors, logistic regression analysis did not identify hypouricemia as an independent predictor of mortality. Instead, a history of malignancy emerged as a strong independent risk factor for mortality, highlighting the complex interplay between malignancy, hypouricemia, and patient outcomes [14].

It can be hypothesized that the catabolic conditions associated with malignancy contribute to increased uric acid clearance, thereby lowering SUA levels in affected individuals. This underscores the importance of maintaining SUA levels within a set physiological range to support overall health, as both excessively high and low uric acid levels have been associated with increased mortality risk [16].

This study adds to the evidence on the complex role of uric acid in chronic inflammatory diseases like BS. The higher incidence of hypouricemia in this cohort compared to the general population, along with its associations with inflammatory markers and clinical features, underscores the need for further research. Future studies should explore the long-term effects of SUA levels on disease progression, treatment response, and overall morbidity and mortality in BS. Such research could improve clinical management strategies and patient outcomes in this complex condition [18].

Following adjustment with IPW, the initially observed associations between hypouricemia and outcomes such as mortality and malignancy were substantially reduced. This suggests that the crude relationships were likely confounded by baseline demographic and clinical variables, rather than representing direct causal effects.

Several limitations of the current study warrant consideration. Firstly, although all consecutive BS patients meeting the inclusion criteria within the study period were analyzed, the hypouricemic patient group (n = 42) fell short of the necessary sample size calculated through our initial power analysis (n = 64). We attempted to address this limitation statistically by employing IPW, which optimizes analytical robustness when group sizes are imbalanced. Secondly, the cross-sectional nature of our research precluded an evaluation of temporal variations in SUA levels and their potential associations with disease activity. Thirdly, the relatively low prevalence of hyperuricemia (3.7%) in our cohort restricted the detailed analysis of its clinical significance within the context of BS. Fourthly, although we excluded those participants receiving medications known to directly affect uric acid metabolism, we could not comprehensively account for all indirect confounding factors, such as dietary habits or the effects of other medications. Lastly, the single-center design of our study may limit the external validity and generalizability of our findings to the broader population of patients with BS.

## 5. Conclusions

In summary, this study demonstrated that hypouricemia is more prevalent among patients with BS compared to the general population. Following rigorous statistical adjustment using IPW to account for imbalanced group sizes and potential confounding variables, the initially observed associations between hypouricemia and various clinical manifestations were substantially attenuated. This analytical refinement effectively addresses concerns regarding the validity of comparisons between unequally sized groups (42 vs. 378) and underscores the value of advanced statistical methodologies in enhancing the robustness of observational research.

### Future Directions

To further elucidate the clinical significance of SUA alterations in BS, future studies should consider employing the following: (1) multicenter cohorts with larger and more diverse populations, particularly to enable a more comprehensive assessment of hyperuricemia, which was infrequent in the present cohort, (2) longitudinal study designs that allow for the serial measurement of SUA levels and their temporal relationship with disease activity and treatment response, (3) mechanistic investigations exploring the molecular pathways linking uric acid metabolism with immune regulation, with a particular emphasis on oxidative stress and antioxidant defense mechanisms, and (4) evaluation of the modifiable determinants of SUA levels, including dietary intake, lifestyle factors, and the use of medications with indirect effects on uric acid homeostasis.

Such approaches would not only overcome the limitations of the current study but also build upon its methodological strengths to advance our understanding of metabolic alterations in inflammatory diseases such as BS.

## Figures and Tables

**Table 1:** Demographic, laboratory, and treatment characteristics of hypouricemic and normouricemic patients with Behçet’s syndrome.

Characteristic	Hypouricemic Patients (n = 42)	Normouricemic Patients (n = 378)	*p*-Value (Before IPW)	Hypouricemic Patients (After IPW)	Normouricemic Patients (After IPW)	*p*-Value (After IPW)
Mean Age (years)	42.8 ± 11.3	43.0 ± 11.6	0.931	42.9 ± 11.4	42.9 ± 11.75	0.873
Mean Disease Duration (years)	12.2 ± 6.1	12.7 ± 8.0	0.603	12.5 ± 6.3	12.6 ± 8.1	0.657
Urea (mg/dL)	23.6 ± 8.9	29.2 ± 19.4	0.065	24.9 ± 9.3	28.3 ± 20.1	0.412
Creatinine (mg/dL)	0.70 ± 0.47	0.80 ± 0.11	0.496	0.73 ± 0.45	0.78 ± 0.10	0.478
Uric Acid (mg/dL)	2.60 ± 0.08	4.66 ± 0.04	<0.001	2.55 ± 0.09	4.62 ± 0.03	<0.001
ALT (U/L)	14.0 ± 1.3	19.0 ± 1.1	<0.001	14.5 ± 1.2	18.8 ± 1.3	<0.001
GGT (U/L)	18.0 ± 5.1	19.0 ± 2.7	0.465	18.3 ± 5.2	19.1 ± 2.9	0.537
ESR (mm/h)	17.5 ± 3.5	18.0 ± 1.0	0.139	17.7 ± 3.6	18.2 ± 1.1	0.467
CRP (mg/L)	3.29 ± 3.80	5.29 ± 1.62	0.219	3.45 ± 3.90	5.11 ± 1.67	0.207
WBC (10^3^/µL)	8.009 ± 4.130	8.795 ± 3.059	0.129	8.11 ± 4.15	8.68 ± 3.10	0.157
Absolute Neutrophil Count (10^3^/µL)	5.737 ± 3.739	5.699 ± 2.658	0.934	5.20 ± 3.80	5.50 ± 2.70	0.312
Neutrophil Percentages (%)	68.2 ± 1.3	62.9 ± 0.5	<0.001	67.0 ± 1.2	61.0 ± 0.7	<0.001
Lymphocytes (10^3^/µL)	1.610 ± 0.531	2.194 ± 0.764	<0.001	1.70 ± 0.56	2.20 ± 0.80	0.017
Neutrophil Lymphocyte Ratio (NLR)	3.54 ± 1.45	2.89 ± 1.89	0.03	3.12 ± 1.40	2.95 ± 2.00	0.201
Hemoglobin (g/dL)	12.6 ± 1.81	13.8 ± 1.77	<0.001	12.7 ± 1.80	13.5 ± 1.60	<0.001
Platelet Count (10^3^/µL)	296,048 ± 107,204	303,324 ± 223,470	0.835	300,758 ± 110,450	303,157 ± 178,430	0.664

Data are presented as median ± SE. ALT: alanine transaminase; GGT: gamma-glutamyl transferase; ESR: erythrocyte sedimentation rate; WBC: white blood cells, TNF: tumor necrosis factor. Note: Values in the “After IPW” columns represent weighted calculations, based on original sample sizes. The IPW methodology assigns weights to subjects according to their propensity scores, creating a balanced pseudo-population where measured covariates are independent of exposure status. This statistical approach strengthens causal inference by minimizing confounding effects while maintaining the original number of observations (hypouricemic n = 42; normouricemic n = 378).

**Table 2:** Gender, clinical manifestations, mortality, and malignancy rates in hypouricemic and normouricemic Behçet’s syndrome patients.

		Uric Acid Level		*p*
		Low (n = 42)	Normal (n = 378)	
Sex	Males	5 (2.4%)	201 (97.6%)	<0.001 ^+^
	Females	37 (17.3%)	177 (82.7%)	
Oral Aphthae	Present	41 (9.9%)	372 (90.1%)	0.703 ^x^
	Absent	1 (14.3%)	6 (85.7%)	
Genital Ulcer	Present	32 (10.3%)	278 (89.7%)	0.711 ^x^
	Absent	10 (9.1%)	100 (90.9%)	
EN	Present	14 (14.1%)	85 (85.9%)	0.116 ^x^
	Absent	28 (8.7%)	293 (91.3%)	
Acneiform Lesion	Present	19 (7.3%)	240 (92.7%)	0.020 ^x^
	Absent	23 (14.4%)	137 (85.6%)	
Pathergy Phenomena	Positive	5 (9.4%)	48 (90.6%)	0.446 ^x^
	Negative	3 (5.6%)	51 (94.4%)	
Vascular Involvement	Present	3 (3.8%)	77 (96.2%)	0.038 ^+^
	Absent	39 (11.6%)	296 (88.4%)	
Uveitis	Present	19 (11%)	154 (89%)	0.574 ^x^
	Absent	23 (9.3%)	224 (90.7%)	
Articular Involvement	Present	8 (7.9%)	93 (92.1%)	0.424 ^x^
	Absent	34 (10.7%)	225 (89.3%)	
Neurological Involvement	Present	4 (10.3%)	35 (89.7%)	0.955 ^x^
	Absent	38 (10%)	343 (90%)	
GI Involvement	Present	4 (23.5%)	13 (76.5%)	0.058 ^x^
	Absent	38 (9.4%)	365 (90.6%)	
MACE	Present	1 (4.2%)	23 (95.8%)	0.494 ^+^
	Absent	41 (10.4%)	355 (89.6%)	
Mortality	Dead	3 (33.3%)	6 (66.6%)	0.018 ^x^
	Alive	39 (9.5%)	372 (90.5%)	
History of Malignancy	Present	3 (42.9%)	4 (57.1%)	0.003 ^x^
	Absent	39 (9.4%)	374 (90.6%)	

EN: erythema nodosum; GI: gastrointestinal; MACE: major adverse cardiovascular events. ^+^ Fisher’s test *p*-value ^x^ chi-square test *p*-value.

**Table 3:** Logistic regression analysis to predict mortality in patients with Behçet’s syndrome.

	Univariate				Multivariate			
Dependent Variable	OR	95% CI		*p*	OR	95% CI		*p*
		Lower	Upper			Lower	Lower	
Gender (male/female)	0.47	0.118	1.94	0.303				
History of Malignancy (Absent or Present)	42.2	8.16	218	<0.001	55.76	6.663	466.67	<0.001
Uric Acid Level (Low or Normal)	0.21	0.0504	0.871	0.032				
Uveitis (Absent or Present)	5.19	1.07	25.3	0.024				
GIS Involvement (Absent or Present)	7.34	1.41	38.2	0.018	11.29	1.639	77.83	0.014
Neurological Involvement (Absent or Present)	0.49	0.0280	8.57	0.329				
Vascular Involvement (Absent or Present)	0.49	0.0611	4.02	0.502				
MACE (Absent or Present)	0.73	0.0419	13.0	0.427				
Chronic Kidney Disease (Absent or Present)	17.10	3.01	97.7	0.001	13.57	1.376	133.81	0.026

Logistic Regression—Backward Stepwise (Wald)/constant: −3.80; OR: 0.02 *p* < 0.001; CI: confidence interval; OR: odds ratio.

## Data Availability

The data utilized in this study were retrospectively collected from hospital information systems and patient medical records following approval by the Institutional Ethics Committee. Due to patient confidentiality and institutional policies, the dataset cannot be made publicly available. However, anonymized data relevant to this research can be made accessible by the corresponding author upon reasonable request, subject to obtaining additional ethical approval where necessary. Requests should be directed to the corresponding author via email at drakaratas@yahoo.com.

## Abbreviations

The following abbreviations are used in this manuscript:

BS	Behçet’s syndrome
ITR-ICBD	International Criteria for Behçet’s Disease
NLRP3	NLR family pyrin domain containing 3
NF-κB	Nuclear factor kappa B
SUA	Serum uric acid
NLR	Neutrophil-to-lymphocyte ratio
ALT	Alanine transaminase
MACE	Major adverse cardiovascular events
OR	Odds ratio
CI	Confidence interval
